# Effect of Corticosteroids on Pain Relief Following Root Canal Treatment: A Systematic Review

**DOI:** 10.22037/iej.2017.26

**Published:** 2017

**Authors:** Foad Iranmanesh, Masoud Parirokh, Ali Akbar Haghdoost, Paul V. Abbott

**Affiliations:** a * Department ofEndodontics, Dental School, Rafsanjan University of Medical Sciences, Rafsanjan, Iran;*; b * Endodontology Research Center, Dental School, Kerman University of Medical Sciences, Kerman, Iran; *; c *Research Center for Modeling in Health, Kerman University of Medical Sciences, Kerman, Iran; *; d *Dental School, University of Western Australia, Perth, Australia*

**Keywords:** Corticosteroid, Endodontics, Flare-Up, Meta-Analysis, Post-Operative Pain, Systematic Review

## Abstract

**Introduction:**

Post-operative pain and flare-up may occur in up to 58% of patients following root canal treatment. The aim was to conduct a systematic review and a possible meta-analysis to determine the effect of glucocorticosteroid (GCS) on pain following root canal treatment.

**Methods and Materials:**

Scopus, MEDLINE and CENTRAL databases were searched up to 30^th^ January 2017 with broad key words. In addition, the reference lists in eligible papers and text books were hand-searched. Assessment of the eligibility of papers and data extraction were performed by two independent reviewers.

**Results:**

Of 9891 articles, 18 were recruited as eligible papers. Most of these papers showed pain reducing effect of GCS on post-endodontic pain. Because of wide heterogeneity among the recruited papers, it was not possible to perform meta-analysis.

**Conclusion:**

Based on the results of this systematic review, there is a vast heterogeneity amongst articles regarding the use of GCS and their effect on post-operative pain after endodontic treatment. Further investigations with similar methods and materials are needed before meta-analysis on the effect of GCS on post-operative pain following root canal treatment can be performed**.**

## Introduction

Oral and dental pain is a popular complaint in the general population [1]. It is generally accepted that as the population grows older, the number of systemic diseases will increase and for that reason the number of patients with more medical complications appearing in dental clinics will increase [2]. One of the main reasons that prevents patients from attending dental offices is anxiety and fear of pain during root canal treatment [1, 3]. For that reason, managing pain during and after root canal treatment is of great importance [4-22].

Pain following root canal treatment (RCT) is undesirable for both the patient and the dental practitioner. The number of patients experiencing pain following RCT has been reported to range from 2.53% to 58% [23-26]. A meta-analysis regarding flare-up following RCT reported an 8.4% (SD±57) incidence of pain and/or swelling among 982 patients from six studies [27]. However, another systematic review and meta-analysis reported that the percentage of patients suffering pain after root canal treatment was dependent on the time span following the treatment. The authors reported 40% of the patients had post-operative pain 24 h after root canal treatment and this decreased to 11% after one week [28].

There is consensus among investigators that the presence of pain following RCT is a multi-factorial phenomenon and there is no single reason for its occurrence [29-37]. Mechanical, chemical, host and microbiological factors have been described as important for inducing pain following root canal treatment [38]. In addition, the presence of pain before treatment, demographic factors (such as gender and age), the pre-operative pulp status, the type of tooth, the type of treatment (initial or re-treatment), and a history of allergy may all influence post-operative pain [29, 39-42]. 

Several strategies have been investigated for pain relief after RCT. These include pharmacological strategies -such as narcotics, analgesics, intra-canal and systemic glucocorticosteroid (GCS), non-steroidal anti-inflammatory drugs (NSAIDs), long action anesthetics, and antibiotics- the use of trephination, occlusal reduction and hypnosis [6, 11, 38, 41-43].

GCS are hormones that have been secreted from the adrenal glands and they have strong anti-inflammatory actions. Several reasons have been outlined for the anti-inflammatory effects of GCS and these include the inhibition of the formation of arachidonic acid from cell membrane phospholipids, the suppression of vasodilatation, the migration of polymorphonuclear leukocytes and phagocytosis [38, 44]. However, because GCS can have side effects, they are not routinely prescribed for systemic use for pain relief following RCT.

Several investigators have used GCS for pain management following RCT [45-76]. However, the findings of most of these studies are not conclusive individually because of their methodological considerations and limitations. 

In the endodontics literature, despite the encouraging results of recent studies [69-76], no systematic review and meta-analysis have been published to answer the debate regarding the benefits of using GCS for post-operative pain. 

Therefore, the aim of this study was to systematically search and review all available published papers regarding the use of CGS in endodontics to assess the effect on pain following RCT.

## Materials and Methods

A broad search was performed to find all published papers reporting clinical investigations regarding the effectiveness of GCS on pain following RCT. 

The search strategy involved defining a clinical PICO question (*P*: problem, *I*: intervention, *C*: comparison, *O*: outcome) as follows: *P*, teeth with inflamed or necrotic and infected pulps having root canal treatment, *I*, GCS prescription, *C*, placebo or the name of materials were used in the control group(s), *O*, incidence of pain after root canal treatment. A wide range of keywords was used to search the databases (Table 1) and the four components of PICO were merged by “AND” in the final step of the search.

In order to be included in this systematic review, articles had to be randomized clinical trials that were published in English between 1^st^ January 1966 and 30^th^ January 2017, and they had to have reported the effect of GCS on pain after RCT. The type of GCS, the type of control (active or non-active), the monitoring period, the route of intervention (intra-canal, parental or oral), sample size, method of pain measurement and pulp status of the teeth needed to be clearly explained.

MEDLINE, CENTRAL, and Scopus databases were searched for the specified period. Citations and references were managed with EndNote version XI. The initial search revealed 9891 citations. After reviewing their titles, abstracts and full texts step by step, 32 articles were selected as being potentially eligible papers [45-76]. The reference lists of these 32 articles, three well known endodontic text books [77-79] and a published study guide [80] were also hand-checked. 

The quality of the 32 articles [45-76] that met the inclusion criteria was assessed using a systematic data extraction sheet constructed by The Critical Appraisal Skills Programme (CASP) (Learning and Development, © Public Health Resource Unit, England 2006) [81] by two independent reviewers. Any disagreements between the two reviewers were checked by a third reviewer during a group meeting. 

Using the data extraction sheet, the first two questions of the questionnaire were screening questions that showed eligibility of the articles. The positive answer to these first two questions showed that the study was a randomized clinical trial with a clearly focused research question. Each positive answer to the next 8 questions had one point. Eligible papers had to have a minimum score of 5 out of 8. Following the use of this questionnaire, 24 articles passed the quality assessment but five of these articles were subsequently excluded [53, 59, 60, 67, 68]. One article [68] was excluded because it was a secondary analysis and had used the data of another investigation [66]; two articles [59, 60] were excluded because they presented their findings in different formats which did not allow the required data to be extracted for the meta-analysis; one study [67] evaluated the pain score following surgery; and another study used the criteria of a flare-up [53] which means pain and/or swelling following RCT that needs an emergency unscheduled visit. The last two studies [53, 67] were also excluded because of their methodological differences compared to the other articles. Therefore, at the end of data analysis, 18 articles were considered for the meta-analysis [46, 47, 50-52, 57, 58, 62, 63, 65, 66, 69-75]. The list of articles and a summary of their findings are shown in Table 2.

## Results

The minimum and maximum sample sizes of the included studies were 37 [52] and 475 [65], respectively. However, the eligible articles used quite different methodologies (Table 2), *i.e.*, the type, route of administration and doses of GCS used, the time intervals and the type of control groups, the method of pain evaluation after endodontic treatment, and the presence of pain before GCS administration. Hence, because of these limitations, the findings could not be aggregated in a meta-analysis.

The discrepancies between the percentages and pain scores in the GCS group versus the control groups were considerably more obvious in the first 48 h after treatment. 

All investigations claimed randomly assigned individuals in either the GCS medication or placebo/active control groups. 

The main finding in the current study was that the route of GCS administration, the dose and type of medication, the type of placebo, the time of pain evaluation following root canal treatment, the method of pain evaluation, the pre-operative pulp status, and the presence of pain before endodontic treatment were quite different in the previously published papers (Table 2) which make it impossible to compare them with each other through a meta-analysis.

## Discussion

The results of this systematic review have shown that use of GCS may reduce pain after RCT and the reduction in pain was much more prominent in the first 12-48 h after treatment. However, because of heterogeneity amongst the eligible papers, it was not possible to perform a meta-analysis on the effect of GCS on pain following endodontic treatment.

Post-treatment pain after RCT is a common problem, particularly for the patients exhibiting pre-operative pain [38]. It has been reported that up to 80% of these patients may experience post-operative pain and the more severe the pre-operative pain then the more severe will be the post-operative pain. Post-treatment pain rarely lasts longer than 72 h and usually is not so severe that it cannot be managed by NSAID agents [44]. 

The present study was designed to search articles in which the effect of post-operative pain after root canal treatment was measured following GCS administration. It should be considered that measuring pain after endodontic treatment will not provide appropriate information regarding the effects of a procedural intervention or the administration of a medication. If there is less pain post-treatment compared to pre-treatment pain, then this may be a sign of appropriate intervention. Moreover, minor transient pain following endodontic treatment that can be easily managed by mild analgesics can be interpreted as a success in post-endodontic pain management in clinical applications. Although consideration of patients who report flare-ups and use of GCS for their pain relief may be a better choice for outcome of the effects of GCS prescription, it was not selected as the O (outcome) in the PICO question of the present investigation because lower flare-up prevalence following endodontic treatment makes it difficult to differentiate amongst the impacts of various interventions on post-operative pain. 

Various classes of medications have been used for pain management following endodontic treatment such as NSAIDs, opioids, acetaminophen and corticosteroids. Previous investigations have confirmed that patients experience their maximum pain during the first 24-72 h following RCT [6, 11, 22, 28, 44]. Results of this systematic review confirm that the major effects of GCS on post-operative pain can be found during this critical early post-operative period following RCT. 

Several limitations prevented the direct combination of the various investigations included in this systematic review. These limitations were a result of differences between the studies and included the route of GCS administration, the type of GCS used, the pre-operative status of pulp and root canal system, the time of GCS administration, the method and time interval of pain evaluation, and the types of controls used. The various differences in methodologies among the studies did not allow their results to be combined and compared. 

Investigations on the effects of GCS on pain after RCT have used various routes of administration for the medications, either by injection (intra-periodontal ligament, supra-periosteal, intraosseous, parental) [46, 57, 58, 69, 72, 74, 75], systemic ingestion [50, 52, 70, 71], or as an intra-canal medicament [47, 51, 62, 63, 65, 66, 73]. All investigations except four have used the medication either as a root canal dressing or as a prescribed medication to be taken after RCT. The three exceptions used GCS as a premedication prior to commencing treatment [70, 71, 75]. Therefore, it was not possible to combine the results of these studies as the route of drug administration may have affected the final outcome. The intra-canal use of GCS may be considered to be safe as only a very small amount of GCS can be inserted into the root canal and therefore there can only be very limited, if any, systemic side effects [82]. In agreement with this, an animal study reported no significant change in corticosteroid plasma level following GCS intra-canal insertion [83]. In addition, placing the active medicament into the root canal enables it to work directly on the inflamed tissues around the apex of the tooth root by diffusing from the canal; in this situation, the root canal acts as a drug delivery system [82]. On the other hand, oral ingestion, and particularly injection, of GCS will produce higher doses of the drug which may provide more anti-inflammatory effects in the periapical tissues and therefore effectively reduce post-operative pain, although the systemic side effects may be greater. In addition, studies that employed systemic GCS [46, 50, 52, 57, 58, 69-72, 74, 75] used known doses of the GCS [44].

Another limitation of performing a meta-analysis was the dose and the type of drug used that made it very difficult to compare the results of the studies. For example, some studies used a long-acting GCS [46, 47, 50, 57, 63, 69, 75], whilst others used various other compositions and forms of GCS [51, 62, 66, 71, 73] (Table 2). Therefore, the results of these studies cannot be directly compared. Meanwhile, the dose of GCS may influence the relief of pain following RCT. Some studies used 4 mg of oral dexamethasone [50, 52, 70], or prednisolone [71] while others used parental forms of dexamethasone [57, 69, 72, 74, 75] or a limited amount of GCS as part of an intra-canal medicament [47, 51, 62, 63, 65, 66, 73]. 

The pre-operative status of the pulp/root canal was a further limitation. Some studies were performed on teeth with inflamed pulps [47, 52, 63, 65, 69, 70, 73-75], others were done on teeth with infected root canal systems and apical periodontitis [62, 66], and some combined the results of treatment for both of these conditions [46, 50, 51, 57, 58, 71-73]. There is no general agreement among investigators that show a correlation between infected root canal systems with apical periodontitis and the presence of pain following RCT [29], although some researchers believe there is a higher incidence of pain following RCT for teeth with infected root canal systems [25, 84, 85].

GCS have potential side effects that should be considered when they are being prescribed [44]. However, two recent investigations reported no side effects [71, 72] associated with a single dose of GCS before RCT. Hence, the concerns of many clinicians may not be justified.

Investigations of the effect of medicaments on pain following root canal treatment compared GCS to either active or placebo medications. Of the 19 articles reviewed in this study [46, 47, 50-52, 57, 58, 62, 63, 65, 68, 69, 70-75], two used calcium hydroxide (CH) as an active control in comparison to GCS [62, 66]. Previous studies on the effect of CH on pain and flare-up following root canal treatment have reported conflicting results. Three studies have reported positive effects of CH in reducing pain after RCT [87-89], but in contrast, three other investigations reported that CH had no significant effect on pain reduction [53, 86, 90]. In accordance with these studies [53, 86, 90] a systematic review and meta-analysis could not show significant impact of CH on reducing post-operative pain following root canal treatment [91]. 

One study compared both types of active and placebo controls with GCS and showed that only Ketorolac had similar pain reduction effects [63]. Ketorolac is a NSAID which has been used for pain control following RCT in several investigations with conflicting results [92-96].

Apart from two studies [62, 63], the other 16 reports included in this systematic review [46, 47, 50-52, 57, 58, 65, 66, 69-75] showed positive effects of GCS on pain relief after RCT when compared to active or passive controls. It has been emphasized that GCS can inhibit or suppress inflammatory reactions and therefore they can control inflammatory mediators that directly or indirectly participate in producing pain [44]. 

The results of the present study show that GCS are much more effective in the immediate post-operative period of time (up to 48 h) following root canal treatment in comparison with longer time periods. This may be due to two main reasons. Firstly, the root canal treatment itself can reduce pain by eradicating the pain stimulants such as pulp tissue remnants, bacteria and their by-products from the root canal system [97].

**Table 1:** Selected Keywords used for the search strategy

	**Keyword**	**For propose**
**Endodontic Procedures**	Pulp/Pulp*	Vital Pulp Therapy/Vital Pulp Treatment(s)/Pulpotomy/Pulpectomy/Dental Pulp Therapy/Dental Pulp Treatment(s)
	Root Canal/Root Canal*	Root Canal Therapy/Root Canal Treatment(s)/Root Canal Obturation
	Endodontic/Endodontic*	Endodontics/Endodontic(s) Treatment/Endodontic(S) Therapy/Endodontic(s) Surgery
	RCT/RCT*	
	Retreatment/Retreatment*	
**Corticosteroid Therapy**	Medication/Medication*	Intracanal Medication(S)/Intra-Canal Medication(S)/Intra Canal Medication(S)/ Root Canal Medication(S
	Dressing/ Dressing*	Root Canal Dressing(S)/Intra Canal Dressing(S)/Intra-Canal Dressing(S)/Intra-Canal Dressing(S)
	Procedure/ Procedure*	Intra-Canal Procedure(S)/Intra-Canal Procedure(S)/Intra-Canal Procedure(S)/ Root Canal Procedure(S)
	Corticosteroid/Corticosteroid*	
	Steroid/ Steroid*	
	Glucocorticoid/Glucocorticoid*	
	**Dexamethasone**	
	**Ledermix**	
	**Prednisolone**	
**Pain**	Pain/Pain*	
	Swelling/Swelling*	
	Flare/Flare*	Flareup(s)/Flare-up(s)/Flare up(s)

Secondly, half of the studies used intra-canal GCS [47, 51, 62, 63, 65, 66, 73] and this allows direct and immediate delivery of the drug to the inflamed periapical tissues which results in rapid pain relief. This has been demonstrated by a study that used a corticosteroid-antibiotic paste as an intra-canal medicament and most of the drug diffused through the apical foramen within the first 3-8 h after treatment [98].

No studies were identified that compared different routes of administration or different doses of GCS and their effects on relief pain after RCT. Therefore, it is not possible at present to recommend one route or dose for GCS although intra-canal delivery does have some advantages as discussed above with respect to safety and efficacy.

Several studies used a combination of an antibiotic and a corticosteroid as an intracanal medicament [62, 65, 66, 73]. It has been emphasized that antibiotics have no significant effect on pain relief after root canal treatment [38, 99] and for that reason it can be assumed that the pain relief following use of GCS provided the therapeutic effect of the medications used in these studies.

Results of the present systematic review were based on published studies with reliable data bases. It is important to note that there may be publication bias regarding GCS effects on pain relief following root canal therapy. In other words, investigations with negative results may not have been published and this may have affected the results of the present meta-analysis. Unfortunately because of the wide heterogeneity amongst the methodologies of these studies, the significance of publication bias using funnel plot and its related statistical tests could not be assessed. GCS have been used for controlling pain and swelling following oral and periodontal surgery [100, 101]. In endodontics, despite the positive effects of GCS that have been reported by several researchers [46, 47, 50-52, 57, 58, 65, 66, 69-75], post-operative pain control strategies are mostly based on the use of systemic NSAID medications [38].

**Table 2.** List of articles included in the review and summaries of their results

**Study **	**TC** *	**Int** †	**Medication**	**SS** **	**Control**	**SS**		**Meas ** §	**Interval**	**R** ¿
**Chance ** ***et al.*** ** [51]**	1	1	Meticortelone©	137	Saline	133		1	24 h	1
**Ehrmann** ***et al.* [66]**	2	1	Ledermix Paste	58	Calcium Hydroxide Paste None	65 71		2	4/24/48/72/96 h	2
**Fava [62] **	2	1	Otosporin	30	Calen Paste (a calcium hydroxide paste]	30		3	48 h/1 w	1
**Glassman ** ***et al.*** ** [52]**	3	2	DecadronΘ	19	Placebo	18		2	8/24/48 h	1
**Kaufman ** ***et al.*** ** [58]**	1	3	Depo-Medrol ۩	18	Mepivacaine 3%	17		4	24 h	1
					None	10				
**Krasner and Jackson [50]**	1	2	Dexamethasone	25	Placebo	23		5	8/24 h	1
**Liesinger ** ***et al.*** ** [57]**	1	4	Dexamethasone	84	Saline	22		6	4/8/24/48/72 h	1
**Rogers ** ***et al.*** ** [63]**	3	1	Dexamethasone	12	Ibuprofen	12		8	6/12/24/ 48 h	1
					Placebo Ketorolac	12 12				
**Marshall and Walton [46]**	1	4	Dexamethasone	25	Saline	25		1	4/24/ 48 h	4
**Mehrvarzfar** ***et al.* [69]**	3	5	Dexamethasone	50	Placebo	50		6	6/12/24/ 48 h	1
**Moskow ** ***et al.*** ** [47]**	3	1	Dexamethasone	26	Saline	24		5	24/48/ 72 h	1
**Negm [65]**										3
**Post-extirpation**	3	1	Kenacomb ʘ	112	Placebo Cream	108		1	1/2/4/8/12/ 24 h	
**Post-instrumentation**				133		122				
**Pochapski ** ***et al.*** ** [70]**	3	2	Dexamethasone	25	Placebo		22	2	4/12/24/ 48h	1
**Jalalzadeh ** ***et al.*** ** [71]**	1	2	Prednisolone	20	Placebo		20	1	6/12/24h	1
**Shaniaee ** ***et al.*** ** [72]**	1	5	Dexamethasone	30	Placebo		30	2	4/8/24/48h	1
**Eftekhar ** ***et al.*** ** [73]**	4	1	Odontopaste Triamcinolone	40 40	Placebo		40	9	24h/7d	2
**Bane ** ***et al.*** ** [74]**	3	6	Methylprednisolone	41	Pulpotomy		43	1	7d/6m	3
**Mehrvarzfar ** ***et al.*** ** [75]**	3	3	Dexamethasone	20	Placebo		20	9	6/12/24/48h	1

* Tooth characteristics - 1: Inflamed and necrotic pulps, 2: Necrotic pulps, 3: Inflamed pulps, 4: Teeth with apical periodontitis.

** Sample size.

† Intervention - 1: intracanal, 2: oral, 3: intra-periodontal ligament, 4: parental, 5: supraperiosteal, 6:Intraosseous

§ Measurement Tool - 1: four categories = without pain, mild pain, moderate pain, severe pain. 2: visual analogue scale (VAS) 0-100 with 4 subcategories = mild, moderate, severe, very severe. 3: three categories = without or mild pain, moderate pain, and severe pain, 4: VAS 0-10 with two subcategories = without pain and painful. 5: VAS 0-100 with three subcategories = mild, moderate, and severe pain. 6: VAS 0-9. 7: VAS 1-10., 8: VAS 0-100 with eight subcategories, 9: Heft-Parker, 10: 5 scale. 4-5: Considered as flare-up.

© Prednisolone acetate 2.5% in an aqueous solution.

® Polymixin B sulfate (10000 IU), Neomycin sulfate (5 mg), and hydrocortisone (10 mg) in an aqueous solution.

‡ Calcium hydroxide (2.5 g), zinc oxide (0.5 g), stabilizing resin (0.05 g), polyethylene glycol 400 (1.75 mL)

۩ Long acting methyl prednisolone

Θ Dexamethasone tablet (4 mg)

ʘ A cream consisting of nystatin, germacidin, and triamcinolone.

¿ R- 1: Randomized without definition, 2: Random table number, 3: Computer generated numbers, 4: Random but with evenly distributed painful teeth in different groups

Fewer side effects of the NSAIDs in comparison to GCS are the most important reasons for selecting the former medication as the first choice for pain relief [38].

## Conclusion

On the basis of this systematic review, heterogeneity in method and materials among the eligible studies make it impossible to perform a meta-analysis. More studies with similar method and materials and the method of evaluation are needed to perform a meta-analysis.
